# *N*-doped TiO_2_ Nanotubes as an Effective Additive to Improve the Catalytic Capability of Methanol Oxidation for Pt/Graphene Nanocomposites

**DOI:** 10.3390/nano6030040

**Published:** 2016-02-26

**Authors:** Xiaohua Wang, Yueming Li, Shimin Liu, Long Zhang

**Affiliations:** State Key Laboratory of Metastable Materials Science and Technology, College of Materials Science and Engineering, Yanshan University, Qinhuangdao 066004, China; wangxiaohua2016@hotmail.com (X.W.); lsm@ysu.edu.cn (S.L.); lzhang@ysu.edu.cn (L.Z.)

**Keywords:** methanol oxidation, TiO_2_ nanotubes, doping, graphene

## Abstract

*N*-doped TiO_2_ nanotubes have been prepared as additives to improve the catalytic capability of Pt/graphene composites in methanol oxidation reactions. Electrochemical experiments show that the catalytic performance of Pt/graphene composites has been greatly improved by the introduction of *N*-doped TiO_2_ nanotubes.

## 1. Introduction

Carbon materials are widely used as electrode materials in supercapacitors, batteries and catalyst carrier in fuel cells [1,2,3]. Novel carbon materials especially such as carbon nanotubes (CNTs), mesoporous carbon, carbon nanoparticles and graphene nanosheets have drawn much more attention in the field of energy storage and conversion due to their high conductivity, large surface area and good chemical stability [4,5].

The direct methanol fuel cell (DMFC) is considered a promising power source for portable electronic devices [6,7]. One key challenge is to develop catalysts with high catalytic capability. The form of carbon is found to play an important role for the catalytic performance of anode catalysts in DMFC [8]. Materials with high surface area will show obvious advantages due to the facilitated mass and charge transport and a higher electrode/electrolyte contact area [9]. Graphene nanosheets have shown improved catalytic performance as support carrier compared with traditional carbon black due to the high specific surface area and high conductivity, according to previous work by us and another group [10,11,12]. However, the catalytic performance for Pt/graphene based catalyst need to be further improved to reduce the usage of noble metal. A lot of methods have been used to improve the electrochemical performance of Pt based catalyst by modifying the support carrier. For example, nitrogen doping has proved to be an effective strategy to improve the electrochemical performance of graphene materials. A series of *N*-doped graphene/Pt nanocomposites have been prepared and showed improved electrochemical activity toward methanol oxidation [13,14,15].

Transition metal oxides such as CeO_2_, SnO_2_, MnO_2_, and TiO_2_ can be employed as support carriers to improve the electrocatalysts activity and stability [16,17]. Among these metal oxides, TiO_2_ is of particular interest due to the good corrosion resistance, low price and environmentally-friendly nature. TiO_2_ as a semiconductor has a low electric conductivity, which limited its total substitution of carbon support carriers. Considering the high conductivity of carbon materials, TiO_2_/carbon materials composites are frequently used as support to overcome the disadvantage of TiO_2_ [18,19]. Furthermore, the electrochemical performance of TiO_2_ is also dependent on its crystal phase and chemical doping [20].

Considering the special properties of *N*-doped TiO_2_, in this report, high performance Pt/graphene catalyst were prepared in which *N*-doped TiO_2_ nanotubes was added as additives, and the catalytic performance toward methanol oxidation reaction indicated that as formed composites had much improved catalytic activity compared with Pt/graphene composites without additives.

## 2. Results and Discussion

**Figure 1:**
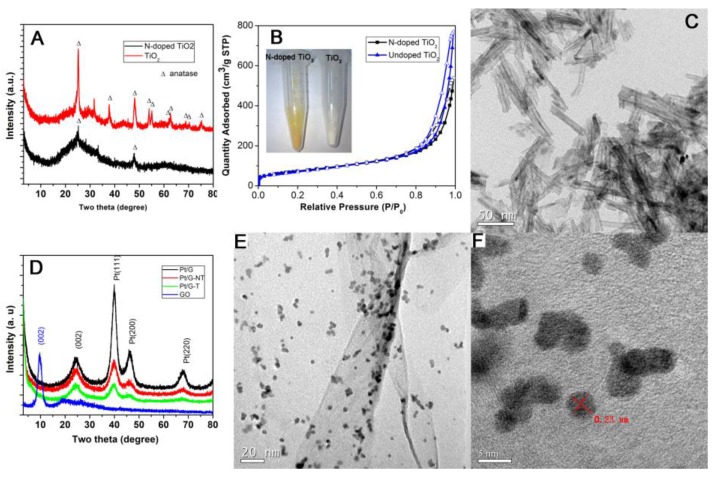
(**A**) X-ray diffraction (XRD) patterns of TiO_2_ nanotubes and *N*-doped TiO_2_ nanotubes; (**B**) N_2_ adsorption/desorption isotherm for *N*-doped TiO_2_ nanotubes and undoped TiO_2_ at 77 K (inset: digital image of *N*-doped TiO_2_ nanotube and undoped TiO_2_ nanotubes); (**C**) Transmission electron microscopy (TEM) images of *N*-doped TiO_2_ nanotubes; (**D**) XRD patterns of graphene oxide (GO, Pt/graphene (Pt/G), Pt/graphene with TiO_2_ (Pt/G-T), and Pt/graphene with *N*-doped TiO_2_ (Pt/G-NT); (**E**) TEM image of Pt/G-NT and (**F**) High-resolution transmission electron microscopy (HRTEM) of Pt/G-NT composites.

Figure 1A shows the X-ray diffraction (XRD) pattern of TiO_2_ nanotubes and *N*-doped TiO_2_ nanotubes. For TiO_2_ nanotubes, most of the strong peaks can be easily indexed to anatase-phase TiO_2_ (JCPDS No. 21-1272). In contrast, the broadened peaks at ~26° for *N*-doped sample might be related to distortion in the O–Ti–O lattice due to the doping of nitrogen into TiO_2_, similar to our previous report [21]. Figure 1B shows the N_2_ adsorption/desorption isotherm at 77 K. These isotherms exhibit characteristics of characteristics of type IV, indicating the presence of meso and micro pores. The total specific surface areas (SSA) of *N*-doped and undoped TiO_2_ evaluated using the Brunauer–Emmett–Teller (BET) equation were 257.7 and 267.6 m^2^·g^−1^, respectively. The *N*-doped TiO2 sample showed a light yellow color, which is obviously different to the white color of undoped sample (inset of Figure 1B). As shown in Figure 1C, the *N*-doped TiO_2_ nanotubes showed the typical hollow tubular morphology with a diameter ~7–10 nm, up to hundreds of nanometer in length, and the wall thickness of the nanotube is approximately 2–3 nm. Notably, TiO_2_ nanorods may form under certain heat treatment of titanium hydrogen oxide nanotubes such as under hydrogen atmosphere [22]. The XRD patterns of the graphite oxide (GO), Pt/graphene (Pt/G), Pt/graphene with *N*-doped TiO_2_ nanotubes (Pt/G-NT) and Pt/Graphene with undoped TiO_2_ nanotubes (Pt/G-T) are shown in Figure 1D. As shown in the XRD pattern of GO, the characteristic peak at 10.8° indicated the successful oxidation of graphite. The strong diffraction peaks at ~39.9° and 46.2° in the XRD patterns correspond to the (111), and (200) facets of the face-centered cubic structures of platinum crystal, which are in good agreement with the cubic Pt (JCPDS No. 4-802). The broad peak located at ~24° indicates the reduction of graphite oxide into poorly ordered graphene nanosheets. Although *N*-doped TiO_2_ nanotubes were added during the preparation process, no peaks corresponding to TiO_2_ were detected due to the small amounts added.

The typical TEM image of Pt/G-NT nanocomposites (Figure 1D) showed that the Pt nanoparticles were deposited on the surface of graphene nanosheets. No TiO_2_ nanotubes additive in the Pt/graphene composites can be seen from the TEM image due to the low ratio of GO: *N*-TiO_2_ nanotubes and the reaction of *N*-TiO_2_ in the solution. As shown in Figure 1E, it is clearly visible that Pt nanoparticles with diameter in the range of 3–10 nm were decorated on the surface of graphene nanosheets. High resolution transmission electron microscopy (HRTEM) investigation (Figure 1F) showed that the lattice fringes with a spacing of ~0.23 nm can be seen in these domains, consistent with the spacing of the (111) planes of Pt calculated from XRD.

**Figure 2:**
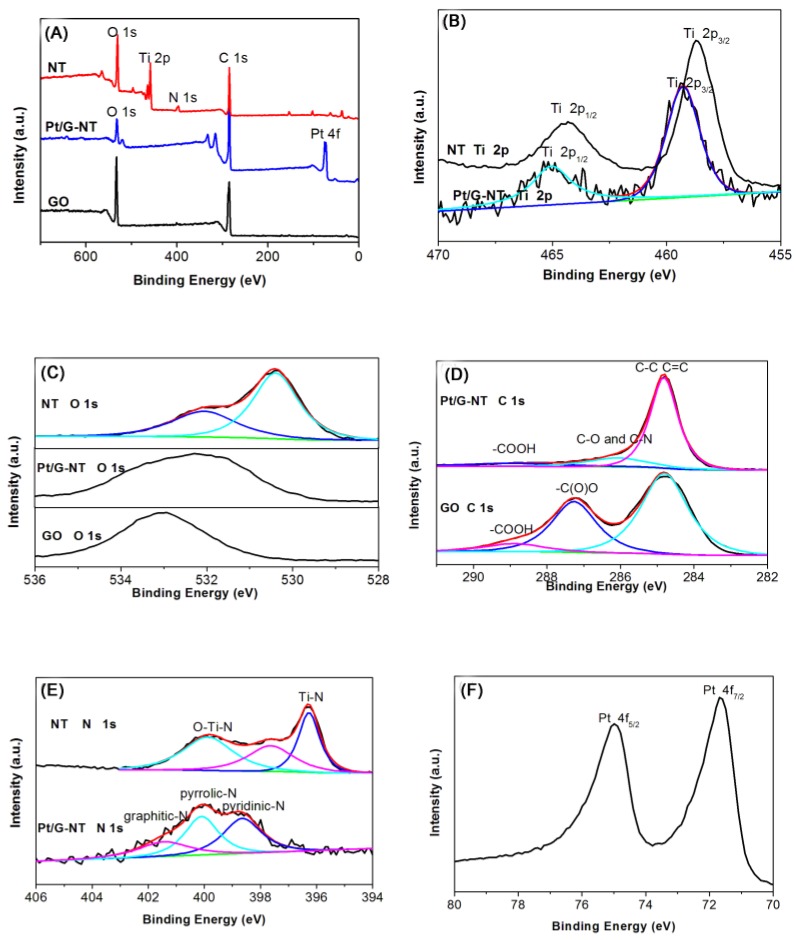
X-ray photoelectron survey spectra of GO, *N*-doped TiO_2_ and Pt/G-NT (**A**), high resolution spectra of Ti 2p (**B**), O 1s (**C**), C 1s (**D**), N 1s (**E**) and Pt 4f (**F**).

The X-ray photoelectron spectroscopy (XPS) survey spectrum of as prepared *N*-doped TiO_2_ (Figure 2A) confirms the presence of Ti, O, and N, while that of Pt/G-NT proves that the presence of Pt, C, O, Ti and N. The Ti 2p XPS spectrum (Figure 2B) can be deconvoluted into three peaks centered at about 284.8, 285.5 and 287.2 eV, corresponding to C–C, C–O and C=O bonding, respectively. Figure 2B illustrates the high-resolution XPS spectra of Ti 2p. Two prominent peaks located at about 458.7 and 464.2 eV for *N*-doped TiO_2_ can be assigned to Ti 2p3/2 and Ti 2p1/2, respectively. The binging energy of Ti 2p peak shifts to lower energies compared to that of Ti^4+^, indicating the presence of trivalent Ti bonding due to the partial substitution of O atom in the TiO_2_ with a N atom [23]. In contrast, the doublet peaks for Pt/G-NT shifted to higher binding energies, suggesting the valence state of Ti^3+^ has changed to Ti^4+^. The entire process is still not very clear. The possible process might be as follows. The “TiN” on the surface of *N*-doped TiO_2_ nanotubes can react with water or NaOH in the solution [24]. During this process, trivalent Ti changed into tetravalent Ti^4+^, while N was released in the form of NH_3_. The as-formed NH_3_ in aqueous solution will react with GO, resulting to the formation of *N*-doped graphene. Quantitative XPS analysis shows that the doping level of Ti in Pt/G-NT is ~0.17 atomic (at.) %. The O 1s spectrum of *N*-doped TiO_2_ is shown in Figure 2C with their peak curve-fitting lines with respect to the chemical states. The O 1s peaks can be fitted into two peaks at about 530.1 and 532.2 eV, which are assigned to Ti–O and chemisorbed oxygen, respectively. In comparison to the C 1s spectrum of the GO, that of Pt/G-NT sample showed sharply decreased intensity for peak(s) corresponding to the epoxy/ether group (286.9 eV, Figure 2D), indicating that most these oxygen containing groups have been removed during reaction. Notably, sp2 C–N peak is overlapping with C–O peak, and sp3 C–N peak is also overlapping with C=O peak [25,26,27]. The high-resolution N 1s XPS spectrum shown in Figure 2E reveals the presence of Ti-N bond as well as O−Ti−N bond (in as prepared *N*-doped TiO_2_ nanotube. The nitrogen content is ~6.47 at % in the *N*-doped TiO_2_ according to quantitative analysis. For Pt/G-NT sample, N 1s spectrum can also be observed. The results indicate that the small amount of N atoms is incorporated into the carbon framework of graphene sheets. Quantitative XPS analysis shows that the doping level of N in Pt/G-NT is ~0.86 at %. As seen from the high resolution N 1s spectrum (Figure 2D), the N 1s peak can be fitted by three component peaks at 398.3, 400.2 and 401.9 eV which can be attributed to pyridinic (N-6), pyrrolic/pyridine (N-5) and quaternary nitrogen (N-Q), respectively. In Figure 2F, the main doublet at 75.1 and 71.7 eV is characteristic of metallic Pt, indicating the reduction of tetravalent Pt.

**Figure 3:**
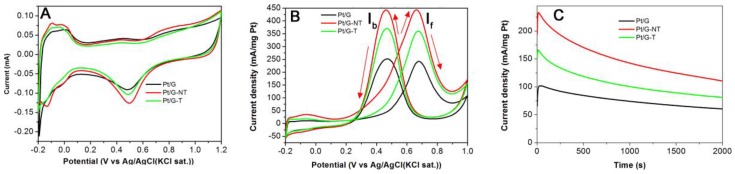
(**A**) Cyclic voltammograms of Pt/G, Pt/G-NT and Pt/G-T an in nitrogen saturated aqueous solution of 0.5 M H_2_SO_4_ at a scan rate of 50 mV·s^−1^. (**B**) Cyclic voltammograms of Pt/G, Pt/G-NT and Pt/G-T an in nitrogen saturated aqueous solution of 0.5 M H_2_SO_4_ containing 0.5 M CH_3_OH at a scan rate of 50 mV·s^−1^. (**C**) Chronoamperometric curves for Pt/G, Pt/G-NT and Pt/G-T catalysts in nitrogen saturated aqueous solution of 0.5 M H_2_SO_4_ containing 0.5 M CH_3_OH at a fixed potential of 0.5 V *vs.* Ag/AgCl (KCl saturated (sat.))

To evaluate the electrochemical activity of as-prepared samples, cyclic voltammogram (CV) experiments were carried out within a potential range from −0.2 to 1.0 V at a scanning rate of 50 mV·s^−1^ in the solution of nitrogen saturated 0.5 M H_2_SO_4_. As seen in Figure 3, the Pt/G-NT electrode shows electrochemically active nature, an obvious hydrogen adsorption characteristic.

The electrochemically active surface areas (ECSAs) were evaluated by integrating the cyclic voltammogram corresponding to hydrogen desorption from the electrode surface [28]. The ECSAs for the Pt/G-NT, Pt/G and Pt/G-T were estimated to be 63, 50 and 58 m^2^·g^−1^ Pt, respectively. It is believed that the high ECSA helps to improve the electrochemical activity of the catalyst.

The electrochemical catalytic activity of the as prepared catalysts toward the methanol oxidation was evaluated by cyclic voltammetric experiments were tested in the solution of 0.5 M CH_3_OH in 0.5 M H_2_SO_4_.

Figure 3 compares the electrochemical catalytic activities of Pt/G-NT, Pt/G-T and Pt/G. For the forward scan, the current (If) increased sharply attributed to the dehydrogenation of methanol and the following oxidation of the absorbed methanol on the electrode sites. The backward peak current (Ib) is related to the subsequent oxidation of the incompletely oxidized products during the forward scan. The ratio of the forward to backward peak current can be used to describe the tolerance of catalyst to the carbonaceous species accumulation [29,30]. As seen in the figure, the ratio of If/Ib for Pt/G-NT is slightly higher than those of Pt/G-T and Pt/G, suggesting that Pt/G-NT exhibits slightly better poisoning tolerance than Pt/G and Pt/G-T.

As seen from Figure 3A, the peak current of Pt/G-NT electrode was 446.8 mA/mg·Pt during the forward potential scanning process, which is much larger than those that for Pt/G-T and Pt/G (~360 and 240 mA/mg·Pt, respectively). Furthermore, the onset potential (where the forward peak current density begins to increase sharply in the CV curve) for Pt/G-NT electrode is also clearly lower than those of Pt/G-T and Pt/G, consistent with a previous report on the introduction of TiO_2_ to Pt based catalyst [31]. Therefore, the performance of Pt/G-NT for the methanol electrochemical oxidation can be considered superior to that of Pt/G or Pt/G-T. As predicted by XPS, the presence of tetravalent titanium oxide is able to enhance dispersion of the wetting process due to the rich active –OH species, enlarging the electrode–electrolyte interfacial area and increasing the concentration of methanol confined around Pt catalyst [16,32,33,34,35]. Furthermore, the improved electrochemical performance is attributed to the *N*-doping effect on graphene nanosheets, because the nitrogen doping can intrinsically regulate the properties of carbon materials in modifying the electronic and chemical properties due to its comparable atomic size and five valence electrons available [36,37,38]. In addition, graphene sheets prepared via GO in the catalysts as an ideal support carrier can provide high surface area, anchor sites to Pt attributed to oxygen-containing groups as well as the high electronic conductivity. Furthermore, the homogeneous dispersion of Pt particles with nano sizes on the 2D graphene nanosheets can maximize the utilization of Pt.

The catalytic stability of the Pt/G, Pt/G-T and Pt/G-NT were examined using chronoamperometry. Figure 3C showed the chronoamperometric curves of 0.5 M CH_3_OH in 0.5 M H_2_SO_4_ solution for these catalyst electrodes at a fixed potential of 0.50 V for 2000 s. It can be clearly observed that the potentiostatic current decreased rapidly at the initial stage for these three electrodes, which might be due to the formation of intermediate species, such as CO_ads_, CHO_ads_, *etc.*, during the methanol oxidation reaction [39]. It is obvious that Pt/G-NT retains the highest current density among these samples during the whole testing time, indicating that the electrocatalytic stability of the Pt/G-NT catalyst for the methanol oxidation was also higher than that of Pt/G-T or Pt/G. The doping of nitrogen into graphene will enhance the electrochemical performance of graphene. The improved electrochemical performance for Pt/G-NT can be attributed to the remaining TiO_2_ and the nitrogen doping of graphene nanosheets.

## 3. Experimental Section

### 3.1. Method

Reagent grade chemicals were analytical purity unless otherwise stated. Graphene oxide (GO) was synthesized from graphite powder based on the modified Hummers method as described elsewhere [40]. Titanium hydrogen oxide nanotubes were prepared by hydrothermal method according to previous report [41,42]. To prepare *N*-doped TiO_2_ nanotubes, as-prepared titanium hydrogen oxide nanotubes were heated in the presence of urea (TiO_2_: Urea, 1:3 mass ratio) at 500 °C for 2 h under Ar atmosphere, yielding yellow powder. The as-prepared product was washed with deionized (DI) water and dried at 100 °C overnight. For comparison, undoped TiO_2_ nanotubes were prepared with the similar condition except that no urea was added during heat treatment, yielding white powder.

To prepare *N*-doped graphene/Pt nanocomposites, GO (80 mg) and *N*-doped TiO_2_ (2 mg) were dispersed in ethylene glycol (EG) (150 mL) and ultrasonic treated for 2 h to form the uniform dispersion solution. Chloroplatinic acid solution (H_2_PtCl_6_·6H_2_O, 5 wt % aqueous solution, 15 mL) was added to the dispersion. The pH of the mixture was adjusted to ~13 by NaOH aqueous solution. NaBH_4_ (100 mg) was slowly added to the dispersion solution. The dispersion was heated at 140 °C for 4 h under magnetic stirring with Ar bubbling. The mixture was then filtered, washed with DI water and ethanol and then freeze-dried overnight, yielding black product, denoted as Pt/G-NT. Pt/graphene nanocomposites with the same Pt loading were prepared with the same procedure except undoped TiO_2_ nanotubes were added, and the final product was denoted as Pt/G-T. For comparison, Pt/graphene with no TiO_2_ added was also prepared via the same procedure, denoted as Pt/G. The loading of Pt for all catalysts is ~40 wt %.

### 3.2. Characterization

The powder X-ray diffraction (XRD) measurements of the samples were recorded on a Bruker D8-Advance X-ray powder diffractometer (Karlsruhe, Germany) using Cu Kα radiation (λ = 1.5406 Å) with scattering angles (2θ) of 10°–80°. A JEOL JEM 2010 transition electronic microscopy (Tokyo, Japan) was used for transmission electron microscopy (TEM) analysis and high-resolution transmission electron microscopy (HRTEM) analysis. The Brunauer–Emmett–Teller (BET) specific surface area was calculated from N_2_ adsorption/desorption isotherms which were obtained by a gas adsorption analyzer (ASAP 2020, Micromeritics Instrument Co. Norcross, GA, USA) at 77 K. X-ray photoelectron spectroscopy (XPS) was carried out on ESCALAB 250XI (Waltham, MA, USA) and the binding energy is calibrated with C 1s = 284.8 eV.

### 3.3. Electrochemical Measurements

Electrochemical measurements were performed on a Princeton P4000 electrochemical working station (Oak Ridge, TN, USA) with a standard three-electrode electrochemical cell. The catalyst electrodes were prepared as follows: 1.0 mg catalyst in 1.0 mL ethanol with Nafion solution was ultrasonicated for 30 min. Then, 5 μL of this suspension was transferred onto a glassy carbon electrode (GC, 3 mm diameter), dried overnight, and used as the working electrode. An Ag/AgCl (saturated (sat.) KCl) electrode was used as the reference and a platinum foil was used as the counter electrode.

## 4. Conclusions

In conclusion, a Pt/graphene nanocomposites catalyst with improved electrochemical performance has been prepared via a facile solution synthesis procedure. The electrochemical experiment proved that the addition of *N*-doped TiO_2_ nanotubes is able to significantly improve the catalytic performance of Pt/graphene composites. The peak current of Pt/G-NT was nearly twice that of unmodified Pt/G catalysts. The stability of *N*-doped TiO_2_ modified catalyst was also much improved compared to unmodified Pt/graphene catalyst, indicating the *N*-doped TiO_2_ nanotube is a very effective additive to modify the electrochemical performance of a Pt-based catalyst.
